# Colorimetric bacteria sensing of *Pseudomonas aeruginosa* using gold nanoparticle probes

**DOI:** 10.1186/s43141-023-00527-4

**Published:** 2023-06-27

**Authors:** Zahra Mousivand, Fatemeh Haddadi, Hossein Kamaladini

**Affiliations:** Department of Biology, Faculty of Sciences, University of Zabol, Sistan and Baluchestan, Zabol, 98613-35856 Iran

**Keywords:** *Pseudomonas aeruginosa*, Gold nanoparticle probes, Colorimetric assay, Multiplex PCR

## Abstract

**Background:**

Due to the advantages of molecular methods over biochemical methods, the use of molecular methods for diagnosing nosocomial infections such as *Pseudomonas* can be an appropriate and rapid way to choose the right diagnosis and treatment of infection and prevent further complications caused by the infection. The present article provides a description of the development of a nanoparticle-based detection technique for sensitive and specific deoxyribonucleic acid-based diagnostic of *Pseudomonas aeruginosa*. Specific thiolated oligonucleotide probes for one of the hypervariable regions of the *16S rDNA* gene were designed and applied for colorimetric detection of the bacteria.

**Results:**

The results of gold nanoprobe-nucleic sequence amplification indicated the probe attached to gold nanoparticles in the presence of the target deoxyribonucleic acid. It caused aggregation of gold nanoparticles in the form of connected networks resulting in color change and indicating the presence of the target molecule in the sample, which could be observed by the naked eye. In addition, the wavelength of gold nanoparticles changed from 524 to 558 nm. Multiplex polymerase chain reactions were performed using four specific genes of *Pseudomonas aeruginosa* (*oprL*, *oprI*, *toxA*, and *16S rDNA*). The sensitivity and specificity of the two techniques were assessed. According to the observations, the specificity of both techniques was 100%, and the sensitivity was 0.5 ng/μL and 0.01 ng/μL of genomic deoxyribonucleic acid for multiplex polymerase chain reaction and colorimetric assay, respectively.

**Conclusions:**

The sensitivity of colorimetric detection was about 50 times higher than the polymerase chain reaction using the *16SrDNA* gene. The results of our study proved to be highly specific with potential use for early detection of *Pseudomonas aeruginosa*.

## Background

Different methods have been developed and applied including immunological assays, nucleic acid probe assays, mass spectrometry, and ejection of areas of the DNA micro-array to detect the target DNA. Detection of the specific DNA sequences paved the way for researchers to develop many diagnostic methods related to target combinations with radioactive fluorescence, chemiluminescent signals, and other types of labeled probes. Direct diagnostic techniques have been established to recognize pathogens and genetic diseases that are dependent on enzymes for producing colorimetric, fluorescent, or light-emitting signals [1]. In recent decades, sensor-based applications that utilize different guidance mechanisms (optical, electrochemical, and sensitive mass measurement) are of great interest in the detection of microorganisms [2]. Biosensing experiments on the basis of gold nanoparticles have been the subject of much research in recent years. They have a diagnostic application due to their simplicity and compatibility [3]. The gold nanoparticle-attached DNA test shows a modern age of biosensor-based diagnostic devices in the biological sciences. They are a major crisis in the development of a sensitive and rapid strategy for detecting pathogen microorganisms [4].

Gold nanoparticles (AuNPs) are highly efficient optical readouts that are widely used for sensitive nucleic acid detection due to their outstanding optical properties that exploit localized surface plasmon resonance [5]. Colorimetric detection using gold nanoparticles could be performed by crosslinking and non-crosslinking methods. Crosslinking mechanism was introduced using a colloidal solution of gold nanoparticles with an approximate size of 5 to 30 nm and red color. The absorption peak of the red solution is about 520 nm, created by plasmin resonance, and the peak moves toward the wavelength when the solution changes to purple [1]. Non-cross-linking DNA hybridization method was described, where the aggregation of the oligonucleotide-functionalized gold nanoprobes is induced by an increasing salt concentration in the presence of complementary/mismatched oligonucleotides of the same size as those of the probe [6]. Synthesized oligonucleotide sequences or PCR-amplified products are required as targets for DNA detection and analysis by both methods.

The application of the DNA-based hybridization method with a thiol attached to gold nanoparticles as a nanoprobe to identify a specific DNA sequence is a growing technique [7] with advantages of being cheap and easy to perform using fluorescence-based or radioactive techniques [8]. Susceptible DNA probe hybridization is a rapid and specific method that has broadly enhanced the detection of bacteria.

Gram-negative *Pseudomonas aeruginosa*, as one of the most problematic pathogens [9], is predominantly dependent on hospital-acquired tuberculosis infections [10] and is very much considered because it is an opportunistic pathogen that causes infection in the immune system [11]. This bacterium is a stimulant for wound infections, urinary tract infections, surgical ulcers, and ear infections [12]. This organism is an intrinsic flora resistant to common antibiotics, even survives in antiseptics, and is a very dangerous and life-threatening bacterium with a relatively high mortality rate of 25–50%. Studies have shown that appropriate antibiotic therapy is associated with reduced mortality. So, this is one of the most important factors for achieving a successful outcome [13]. *P. aeruginosa* infections are widely recognized by standard microbiological methods like phenotypic and biochemical profiles; however, these conventional tests have important drawbacks including long-time detection, the need for special equipment, and being unreliable [14, 15].

An accurate and rapid system for detecting *P. aeruginosa* in isolated patients and preventing further spread of the disease is very important. Bacterial culture is one of the most important techniques which is still being used in diagnostic microbiology due to its ability to limit the viable bacteria in the sample as well as obtain a pure sample for further testing [16]. Many studies have used the API 20e system or traditional biochemical testing to detect bacteria [17], although the adaptability of *P. aeruginosa* has caused problems with these methods. Therefore, it is essential to develop specific genetic systems that can accurately detect this microorganism [18].

One of the fast and reliable molecular techniques to detect microbial pathogens is PCR [19]. Based on this method, various diagnostic PCR-based assays have been developed for the identification of *P. aeruginosa*. The separation of *P. aeruginosa* strains shows patients with excessive genotype diversity and several studies have shown that one or more virulence genes in some *P. aeruginosa* strains are low. Several PCR protocols have been reported to overcome these problems, but there is a genetic exchange between *P. aeruginosa* and almost all the bacteria associated with it; hence, most of the applied methods have low specificity. Therefore, there is a need to introduce and develop a comprehensive and reliable PCR for the detection of *P. aeruginosa* [20].

The present study is an attempt to optimize a multiplex PCR targeting four different specific genes for *P. aeruginosa* (*oprL*, *oprI*, *toxA*, and *16S rDNA*) simultaneously for comprehensive and confirmatory identification. In addition, a colorimetric assay using thiolated probes of *16S rDNA* gene was applied for the detection of *P. aeruginosa*. Consequently, the detection limit, sensitivity, and specificity of the assays were tested.

## Methods

### Bacterial strains and DNA preparation

*P. aeruginosa* (ATCC 27853) strain was provided by Bu-Ali Hospital. The bacteria listed in Table 1 were applied to assess the specificity of the test. Bacteria culture was performed in LB (Luria-Bertani) broth (Merk, Germany) and grown overnight at 37 °C in an incubator shaker at 200 rpm. DNA was isolated using a simple and rapid boiling procedure, and the genomic DNA was stored at − 20 °C until use.

**Table 1:** List of bacterial strains

Bacteria	Strains	Control
*Pseudomonas aeruginosa*	ATCC 25922	+
*Escherichia coli*	ATCC 25922	−
*Staphylococcus aureus*	ATCC 25923	−
*Enterococcus faecalis*	ATCC 29212	−
*Acinetobacter baumannii*	ATCC19606	−
*Staphylococcus epidermidis*	ATCC 12228	−

### Primer design

The sensitivity and specificity of *P. aeruginosa* detection were investigated using 4 specific genes *toxA*, *oprL*, *oprI*, and *16S rDNA*. The full sequence of genes *toxA* (GI: JX026663.1), *oprL* (GI: Z50191.1), *oprI* (GI: X58714.1), and *16S rDNA* (GI: AB037545) was obtained from the NCBI database to design primers using MP-Primer (http://biocompute.bmi.ac.cn/MPprimer/) (Table 2). The characteristics of the designed primers were evaluated and verified via Integrated DNA Technologies available at (http://www.IDTDNA.com) and NCBI BLAST database. Ultimately, the primers’ sequence was sent to the MACROGEN Korea Company for synthesis.

**Table 2:** Designed primers used in multiplex PCR

Genes	Primers	Sequences (5′–3′)	Amplicon size (bp)	Annealing temperature (°C)	Reference
*toxA*	TOA-F	ACGCCCTGCATGTATCCTCCGA	521	62	
	TOA-R	CTTCGAGGCGGATGGTCAG			
*OPRL*	OPL-F	TAGTGCTGGAAGGCCACACCGA	256	62	
	OPL-R	AGGAACGTCAGGACACGCAGGT			
*OPRI*	OPI-F	AAACCGAAGCTCGTCTGACCGC	172	62	
	OPI-R	TTGCGGCTGGCTTTTTCCAGCA			
*16S rDNA*	16S-F	GGGGGATCTTCGGACCTCA	956	62	[31]
	16S-R	TCCTTAGAGTGCCCACCCG			

### Multiplex PCR

The multiplex PCR was performed in a total volume of 50 μL containing the following components: 1X PCR buffer II; 0.4 mM (each) deoxynucleoside triphosphate; 0.4 mM of each TOA-F and TOA-R, also 0.2 mM of OPL-F, OPL-R, OPI-F, OPI-R, and 16S-F, 16S-R, primers, 1.25U of AmpliTaq; and approximately 5 ng of template DNA. A preliminary study on gradient PCR was done using different temperatures at 57–63 °C, and finally, 62 °C was selected as an optimum annealing temperature to carry on the multiplex PCR. PCR amplifications were performed in a DNA Thermal Cycler 480 (Applied Biosystems, USA) with the following parameters: predenaturation for 4 min at 94 °C; 30 cycles of 94 °C for 30s, 62 °C for 90 s and 72 °C for 90 s; and post-extension for 7 min at 72 °C. PCR products (10 μL) were run on 1% agarose gel at 90 V and visualized with ethidium bromide.

### Colorimetric detection of *P. aeruginosa*

#### Alkanethiol-modified oligonucleotides and preparation of DNA

Specific probes amplifying the *16S rRNA* gene sequence across the internal regions of the target DNA of the *P. aeruginosa* were designed. The 5′-(alkanethiol)-capped probe p5thiol [Au-NPs-SH-(CH2) 6-5-GAGCTAGAGTACGGTAGAGGG] and 3′-(alkanethiol)-capped probe p3thiol [GGTGGAATTTCCTGTGTAGCG-3-(CH2) 3-SH-Au-NPs] were synthesized by BIONEER Company, South Korea.

Preparation of DNA was performed by PCR amplification of *16SrDNA* using the protocol and program described in the “Multiplex PCR” section.

#### Preparation and hybridization of thiol-modified probes with DNA

**Fig. 1:**
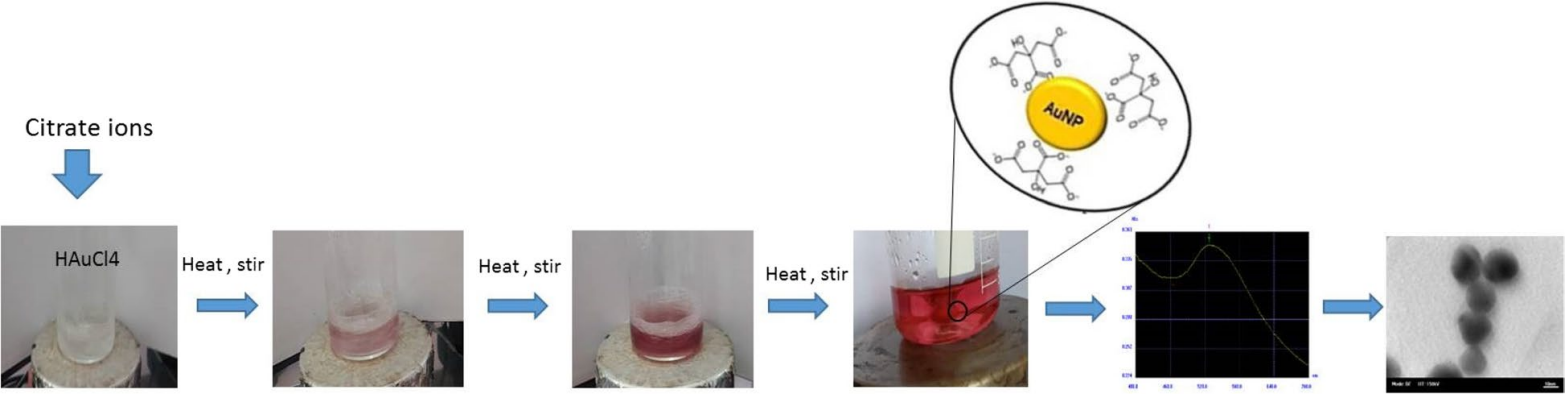
Preparation and characterization of Au nanoparticles

**Fig. 2:**
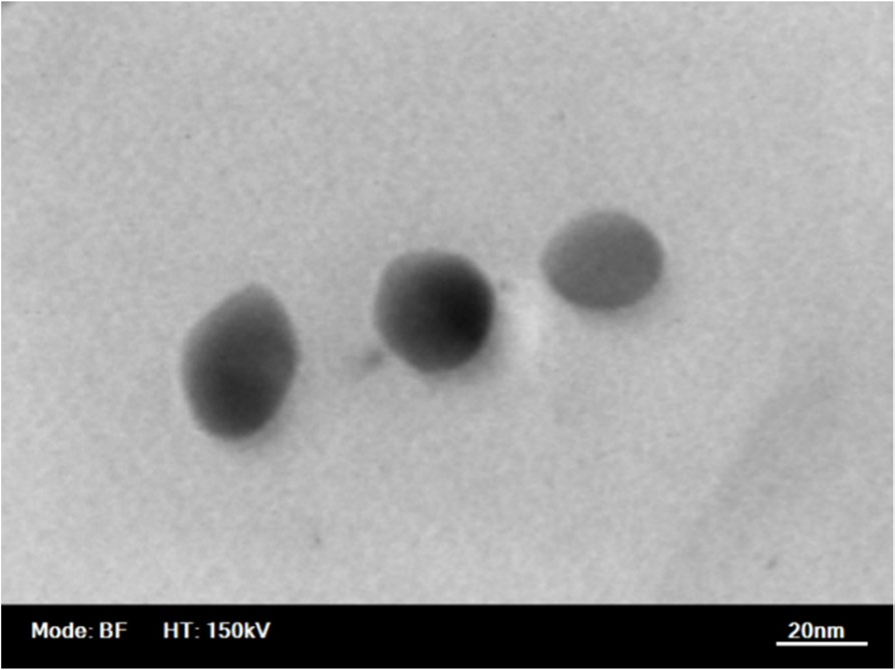
TEM electron microscope image of gold nanoparticles

Au colloids were prepared by the Katherine method with minor modifications by citrate reduction of HAuCl_4_ (Sigma-Aldrich, USA) (Fig. 1). A typical solution of 20 nm diameter gold particles exhibited a characteristic surface plasmon band centered at 518–520 nm. Transmission electron microscopy (TEM) with a Hitachi 8100 transmission electron microscope was used to determine the size and monodispersity of the resulting nanoparticles (Fig. 2).

DNA probes were modified with a thiol group at its 3′ or 5′ end to facilitate their conjugation on the surface of the gold nanoparticles (GNPs). The probes were prepared by conjugating the thiol DNA probe and GNPs (Hill and Mirkin, 2006). The preparation of DNA was performed by PCR amplification of *16SrDNA*. DNA hybridization was performed using two complementary 21-mer DNA molecules according to the method suggested by Gill et al. [22]. After DNA denaturation at 95 °C for 5 min and ice chilling for 5 min, the prepared single-stranded DNA was added to 100 μL of solution containing gold NP probes. In this solution, there was a final concentration of 3.6 pmol of each probe. After being mixed at 37 °C, the solution was immediately measured by colorimetric method using UV/visible spectrophotometer (Rayleigh UV 2100, China).

#### Determination of the multiplex PCR and colorimetric assay specificity and sensitivity

Specificity of multiplex PCR and colorimetric assay for the detection of *P. aeruginosa* were performed using the *P. aeruginosa* DNA and DNAs of *Escherichia coli* (ATCC 25922), *Staphylococcus aureus* (25923), *Acinetobacter baumannii* (19606), *Staphylococcus epidermidis* (12228), and *Enterococcus faecalis* (29212) as negative control bacteria species. In addition, the sensitivity of multiplex PCR and gold NP-bound probe hybridization was determined by testing various genomic DNA concentrations of *P. aeruginosa* using dilution rage from 200 to 0.01ng/μL to determine the limit of detection of the optimized methods. In addition, to compare the limit of colorimetric detection and PCR, PCR was performed using only the *16S rDNA* primers and a gradual reduction of DNA concentration from 200 to 0.01 ng/μL.

## Results

### Multiplex PCR detection of *P. aeruginosa*

**Fig. 3:**
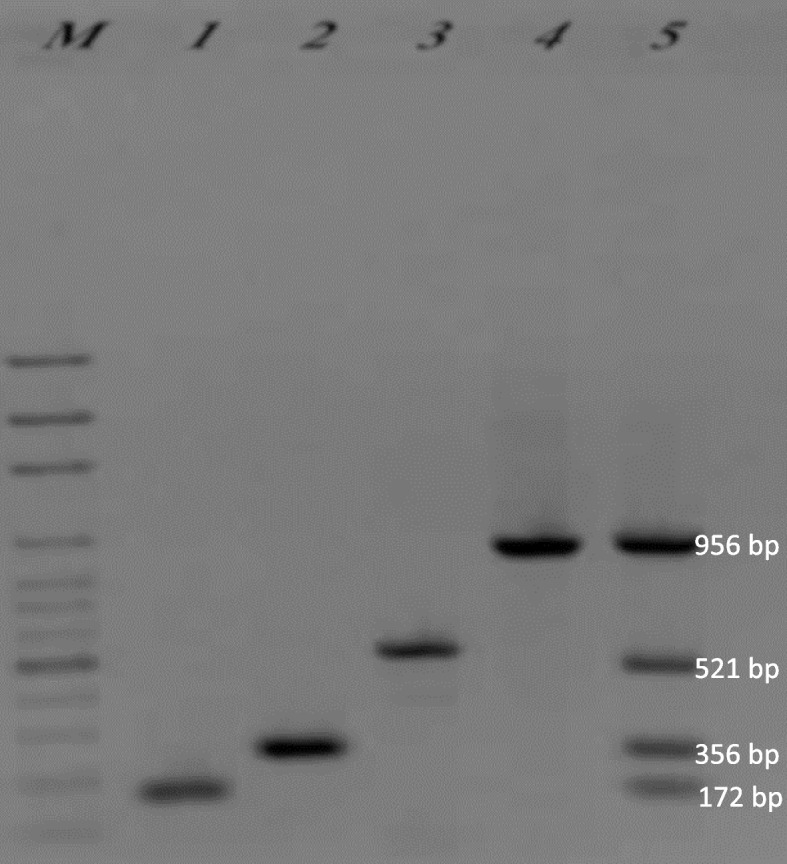
PCR amplification products. M, ladder; 1, *oprI* gene; 2, *oprL* gene; 3, *toxA* gene; 4, *16S rDNA* gene; 5, multiplex PCR performed with primers of the four genes

Amplified PCR products of 172, 256, 521, and 956 bp corresponding to *oprI*, *oprL*, *toxA*, and *16S rDNA* genes were observed, respectively (Fig. 3). The best annealing temperature was 62 °C for each of the four genes. The results of multiplex PCR using four pairs of primers indicated that the four fragments corresponded to all of the four genes confirming the accuracy of the annealing temperature.

**Fig. 4:**
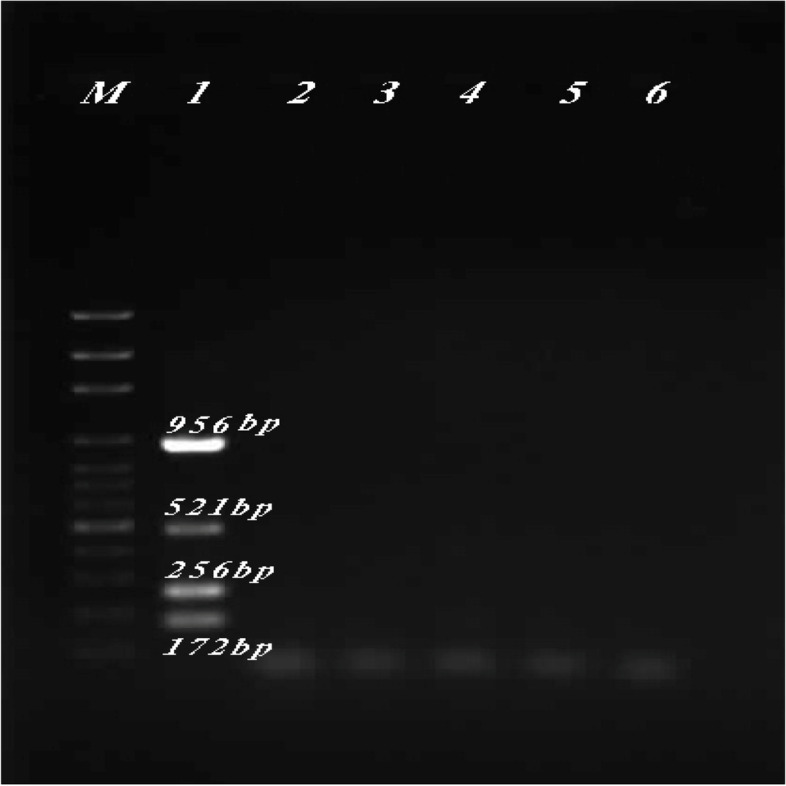
Determination of the designed primer specificity. M, ladder; 1, *P. aeruginosa* (27853); 2, *Escherichia coli* (ATCC 25922); 3, *Staphylococcus aureus* (25923); 4, *Acinetobacter baumannii* (19606); 5, *Staphylococcus epidermidis* (12228); 6, *Enterococcus faecalis* (29212). Expected fragments corresponded to the primers of the four genes only amplified in the presence of the *P. aeruginosa* (27853) genomic DNA

Multiplex PCR reactions with DNA of negative control bacteria species including *Escherichia coli* (ATCC 25922), *Staphylococcus aureus* (25923), *Acinetobacter baumannii* (19606), *Staphylococcus epidermidis* (12228), and *Enterococcus faecalis* (29212) showed no amplified fragment corresponding to the designed primers (Fig. 4). The expected fragments were only amplified in the presence of *P. aeruginosa* genomic DNA, confirming the specificity of the designed primers for the four genes.

**Fig. 5:**
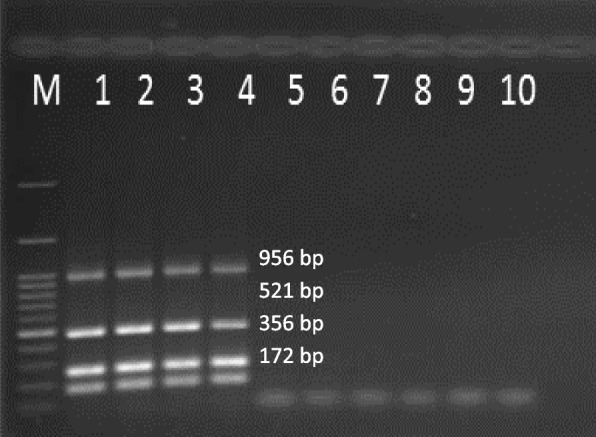
Determination of the multiplex PCR sensitivity using different concentrations of *P. aeruginosa* genomic DNA. M, ladder; 1, 200 ng/μL; 2, 100 ng/μL; 3, 50 ng/μL; 4, 20 ng/μL; 5, 10 ng/μL; 6, 1 ng/μL; 7, 0.5 ng/μL; 8, 0.1 ng/μL; 9, 0. 05 ng/μL; 10, 0.01 ng/μL

To determine the sensitivity of the designed primers, different concentrations of *P. aeruginosa* DNA were tested, and the results showed four bands corresponded to the four primers from 200 to 20 ng/μL (Fig. 5). Concentrations lower than 20 ng/μL did not show all the fragments; hence, the least concentration limit for the detection of the bacteria using the four pair of primers was 20 ng/μL.

### Colorimetric detection of *P. aeruginosa*

**Fig. 6:**
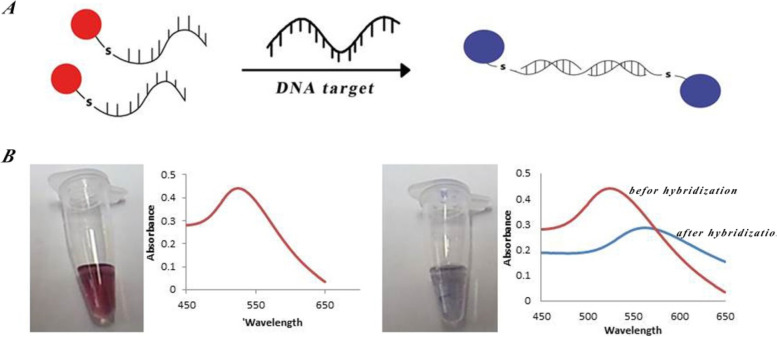
A brief description of the purpose of identifying the DNA by gold nanoparticles probe. **A** When two oligonucleotide probes labeled with gold are attached to the target, it causes the accumulation of gold nanoparticles. **B** When the target DNA is not present and the solution remains red, the spectrophotometer analysis shows the maximum absorption at 524 nm. In the presence of the target DNA, gold nanoparticles accumulate. The hybridization between the probe and the target DNA leads to a change of color from red to violet and reduces absorption at the wavelength of 558 nm

Colorimetric detection of *P. aeruginosa* by the thiol-capped oligonucleotide probes bound to GNPs showed a change in color and wavelength in the reaction mixture. Wavelength changes were investigated between 400 and 600 nm before and after the hybridization of gold NP-bound probes with target DNAs (extracted from *P. aeruginosa*) (Fig. 6). The wavelength changes from 524 to 558 nm could help to distinguish whether or not the probes are attached to the target DNA. Following the addition of the probes, a color change was observed. It is suggested that a specific region leads to the aggregation and density of gold NP-bound probes on the sequence.

### Specificity and sensitivity determination of gold NP-bound probes hybridization with target DNA

**Fig. 7:**
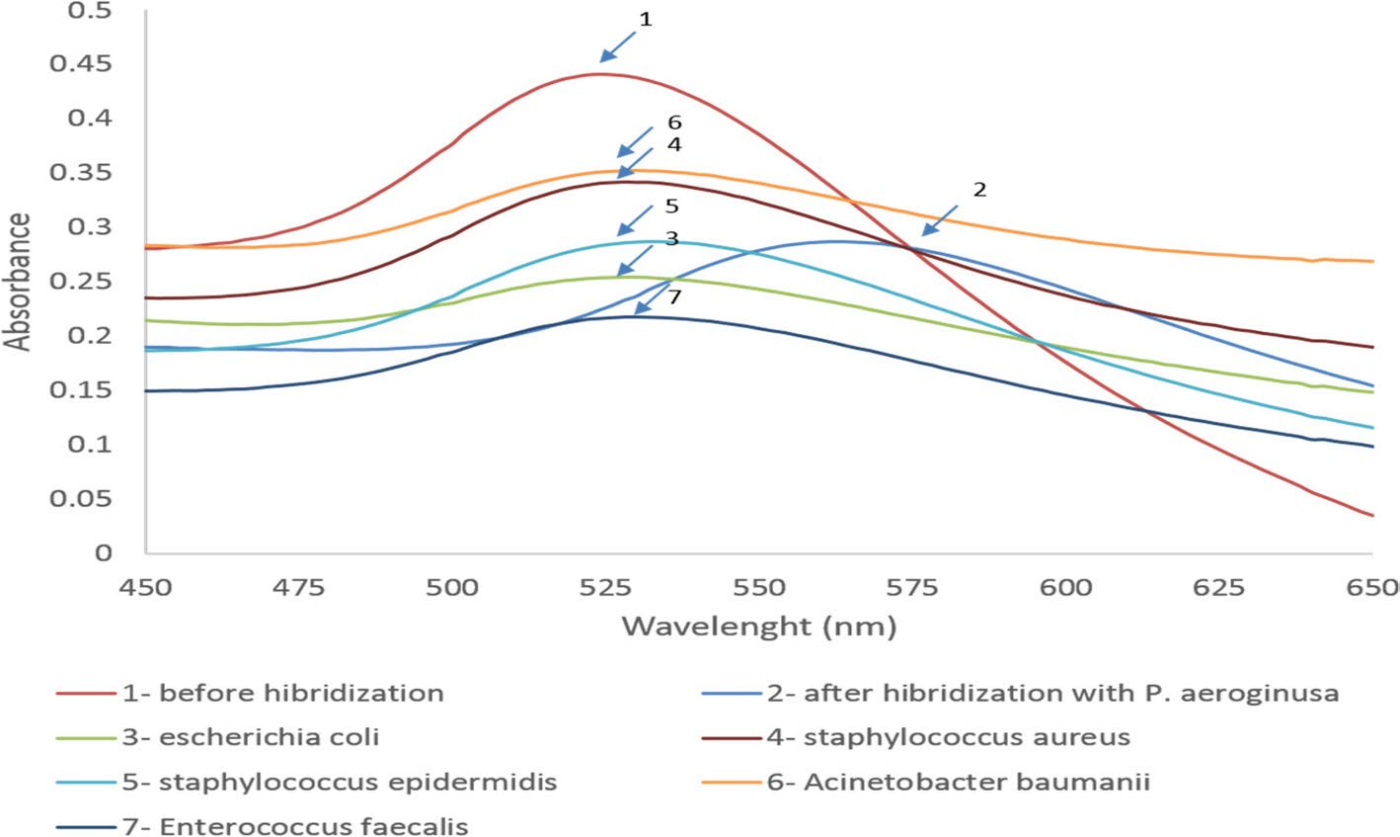
The absorbance spectrum obtained before (1) and after (2–7) adding the gold NP-bound probes to the DNA isolated from *P. aeruginosa* (2) and bacterial species selected as negative control (3) *Escherichia coli*, (4) *Staphylococcus aureus*, (5) *Staphylococcus epidermidis*, (6) *Acinetobacter baumannii*, and (7) *Enterococcus faecalis*). Adding the gold NP-bound probes to the DNA of negative control bacteria did not lead to any alteration in maximum absorption length at 524 nm

To assess the specificity of Gold NP-bound probes of standard strains, DNA of *Escherichia coli*, *Staphylococcus aureus*, *Enterococcus faecalis*, *Acinetobacter baumannii*, and *Staphylococcus epidermidis* were applied as negative controls. Figure 7 exhibits the average absorbance spectrum for negative controls before and after adding the gold NP-bound probes. As shown in Fig. 6, there was no alteration in the absorbance spectrum suggesting the lack of target sequence of the *16S rDNA* gene in DNA isolated from negative control bacteria. These bacteria do not possess the *16S rDNA* gene sequence in their genomes. For this reason, adding the gold NP-bound probes to their DNA did not trigger hybridization between the probe and the bacterial DNA, and as a consequence, no change in wavelength was observed.

**Fig. 8:**
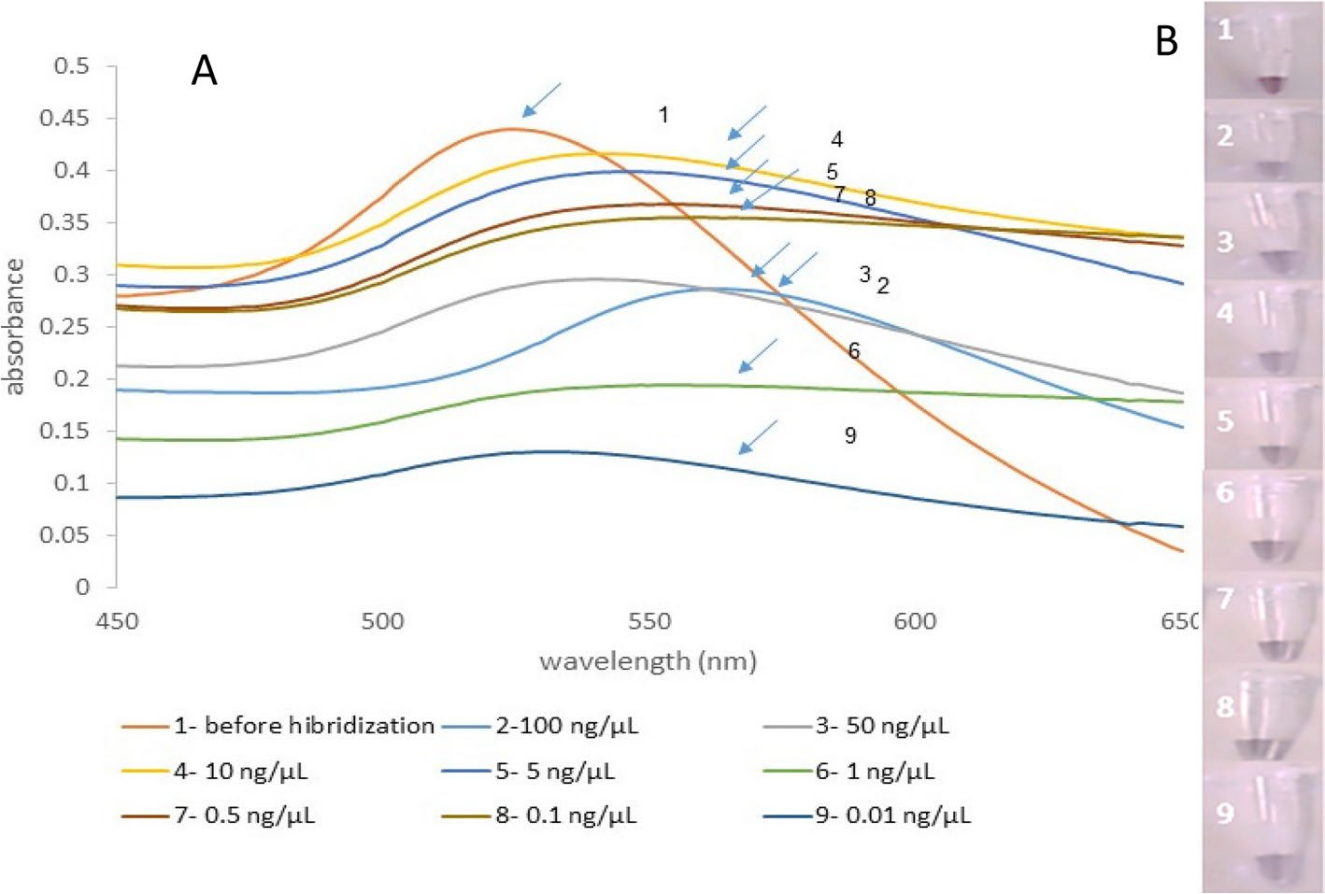
**A** Sensitivity determining of the gold NP probes hybridization with *P. aeruginosa* DNA using different concentrations of bacterial genomic DNA. 1, before hybridization; 2, 100 ng/μL; 3, 50 ng/μL; 4, 10 ng/μL; 5, 5 ng/μL; 6, 1 ng/μL; 7, 0.5 ng/μL; 8, 0.1 ng/μL; 9, 0.01 ng/μL. **B** The gold nanoparticles’ color changed by increasing DNA concentrations

**Fig. 9:**
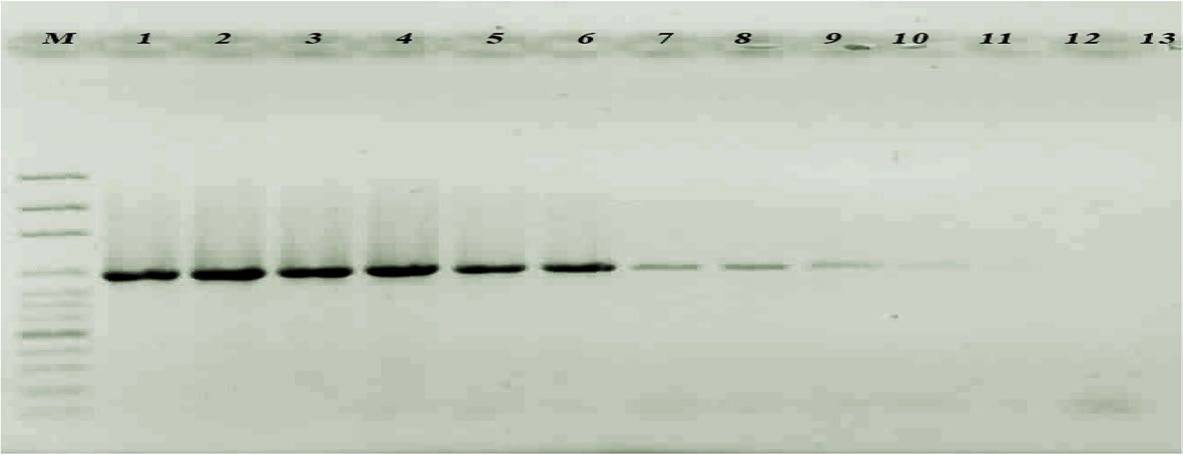
Sensitivity of the 16S rDNA PCR with different concentrations of genomic DNA. M, ladder; 1, 200 ng/μL; 2, 100 ng/μL; 3, 50 ng/μL; 4, 20 ng/μL; 5, 10 ng/μL; 6, 5 ng/μL; 7, 1 ng/μL; 8, 0.5 ng/μL; 9, 0.01 ng/μL; 10, 0.005 ng/μL; 11, 0.001 ng/μL; 12, 0 ng/μL

By gradual reduction of the genomic DNA concentration from 200 ng/μL, a change in color was observed at 0.01 ng/μL of genomic DNA (triplicate results) (Fig. 8). Hence, the sensitivity of hybridization was 0.01 ng/μL of genomic DNA. The results of PCR using 16S rDNA showed a higher limit of detection compared to the colorimetric detection, and the method was able to detect the bacteria up to 0.5 ng/μL (Fig. 9).

## Discussion

The application of sensor-based methods to detect pathogenic agents comes into consideration in recent years. Identification of *P. aeruginosa* as one of the most important nosocomial infections, from clinical samples by traditional culture methods, usually takes more than 2 days; hence, molecular identification of the pathogen was considered to develop fast, sensitive, reliable, and accurate detection methods [21, 22]. In the present study, we can detect *Pseudomonas aeruginosa* within 4 h by colorimetric assay. Simplicity, practicality, and cost-effectiveness of the colorimetric assays provide the opportunity for rapid, sensitive, selective, and convenient detection of bacteria simply with the naked eye and help in the early detection of pathogens, hence minimizing social and economic losses. Colorimetric detection methods as a platform in biosensor development have been applied widely using nanomaterials like nanoparticles, nanorods, and nanowires. Gold (Au), silver, and copper are the most common nanoparticles applied to develop biosensors. Among the established assays, gold nanoparticle probes are suggestive to detect a specific region of the pathogen target gene; hence, in this study, we used gold nanoparticles to develop the colorimetric assay. Besides high sensitivity and specificity, gold nanoprobe assay is a simple and fast way of detecting pathogens in the environment and pathogens in critical situations. Also, it could be applied as one of the economical assays using molecular diagnostic techniques. One of the main advantages of this detection is the achievement of the results with no specific instruments and advanced diagnostics through color change with the naked eye using either “aggregation” or “non-aggregation” detection strategies [22]. In the current study, to develop gold nanoprobe assay, the *16SrRNA* gene was due to its high specificity for the detection of pathogens by targeting highly conserved regions of the *16SrRNA* gene.

Here, the gold nanoparticle probe assay mainly includes two steps of *P. aeruginosa* DNA amplification by PCR and addition of gold nanoparticle probes for detection. The gold nanoparticle probes were added to the heat-denatured *P. aeruginosa* PCR products and were incubated at 55 °C for 2 h for DNA hybridization. The absorbance of the solution was estimated by a spectrophotometer. When one-stranded target DNA of *P. aeruginosa* was available in the solution, the gold nanoparticle probes aggregated through hybridization to the target DNA leading to reduced absorbance of the solution at 525 nm and changing the suspension color from red into reddish purple. In contrast, the color and absorbance pattern did not change if the specific target DNAs of *P. aeruginosa* were missing in the solution. In spite of the fact that color movement may well be recognized by spectrophotometry as early as 2 h after the expansion of probes, a noteworthy difference can be observed at approximately 4 h by direct observation.

An assessment of gold nanoparticles for detecting bacteria and viruses has been previously reported. A nanoparticle-based method is used for detecting *Helicobacter pylori* with 98% sensitivity and specificity [23], *Escherichia coli* with a detection limit of 2.17 pM of target DNA [24], pirAvp toxin gene (causative agent of acute hepatopancreatic necrosis disease) at 20 fg/µL of shrimp genomic DNA [25], influenza virus with a detection limit of 10 ng/ml [26], *Salmonella* with 89.15% sensitivity and 99.04% specificity [27], *Mycobacterium tuberculosis* (MTB) with 96.6% sensitivity and 98.9% specificity, *Mycobacterium tuberculosis* complex (MTBC) with 94.7% sensitivity and 99.6% specificity [28, 29], *Pseudomonas syringae* pathovars with a sensitivity as low as 15 ng/mL of genomic DNA [2], *S. epidermidis* with a detection limit of 20 ng/mL [18], and *Klebsiella pneumoniae* with a sensitivity of 1 pg/μL [30].

We preformed the colorimetric assay using amplified fragments of *16S rDNA* using PCR; however, there is an opportunity to use direct target DNA extracted from bacteria and the probe combination with gold nanoparticles, making this method easy, fast, and inexpensive. Nonetheless, the application of PCR products can increase the accuracy of the assay. Moreover, reducing the concentration of genomic pattern for hybridization determines that a very small genomic pattern concentration can help us to detect *P. aeruginosa* in the gold nanoparticle method.

The previous studies were performed on different genes such as *16S rDNA*, *gyrB*, *oprL*, *ETA*, *algD*, *chitA*, and *fliC* either individually or in combination, for different objectives using different methods. Due to the high genetic diversity of the *P. aeruginosa* species, some genes have lower sensitivity (e.g., *toxA*) which caused false-negative results. Hence, we have selected four specific primer pairs of *P. aeruginosa* genes for the detection of the bacteria using multiplex PCR, to eliminate the chances of false-positive results of closely related species with target *P. aeruginosa* bacteria*.* The results showed sensitivity of multiplex PCR-based test in diagnosing genomic DNA showed the minimum required concentration of 20 ng/μL. According to the results, multiplex is less sensitive; however, given the simultaneous use of four specific genes, this method is acceptable. The sensitivity of PCR in the detection of *P. aeruginosa* based on the *16S rDNA* gene was 0.5 ng/μL, and gold nanoparticle probes were 0.01 ng/μL of the genomic DNA, indicating that the sensitivity of the colorimetric assay is 50 times higher than that of PCR in the identification of *P. aeruginosa*. The higher sensitivity and specificity obtained in this study using gold nanoparticle probes is in accordance with the findings of the previous studies by Gill et al. [23] on *Helicobacter pylori* and Vasheghi et al. [2] on *Pseudomonas syringae*.

The gold nanoparticle probe represents a specific region that causes the accumulation of probes attached to gold nanoparticles on the target sequence. There are a number of important factors that modulate the probe’s specificity to the target DNA. These include the size of the gold nanoparticles, the temperature of the hybridization, the amount of GC, the length and arrangement of probes, and the type and concentration of salt. We used the most optimal values related to this parameter, including the thickness of about 20 nm, the temperature of hybridization at 50 °C, the 70% GC of the probe and placement of head-to-head, and a concentration of one mole of salt according to the parameters reported by [31–33]. All different aspects of the binding reaction can undoubtedly affect the success of the combination. We tried to use the most optimal factors to increase the probability of binding the probe to the target DNA.

## Conclusion

Although the GNP and multiplex-based PCR techniques are currently widely recognized in molecular and immunological diagnosis, few reports suggest compressing both techniques in a test to quickly diagnose specific DNA sequences. According to our findings, colorimetric detection of *P. aeroginusa* could be a reliable technique for the direct diagnosis of clinical specimens with sensitivity and specificity compared to conventional or commercial systems. The optimized colorimetric assay was also indicated to be fast, simple, and inexpensive with a sensitivity of 50 times higher than the PCR.

## Data Availability

The datasets used and/or analyzed during the current study are available from the corresponding author upon reasonable request.

## Abbreviations

DNA: Deoxyribonucleic acid
API: Analytical profile index
PCR: Polymerase chain reaction
LB: Luria-Bertani
PBS: Phosphate-buffered saline
NCBI: National Center for Biotechnology Information
BLAST: Basic Local Alignment Search Tool
DTT: Dithiothreitol
TEM: Transmission electron microscopy
NP: Nanoparticle
GNPs: Gold nanoparticles
SDS: Sodium dodecyl sulphate
NaCl: Sodium chloride
UV: Ultraviolet
Au: Gold
MTB: *Mycobacterium tuberculosis*
MTBC: *Mycobacterium tuberculosis* complex
